# Functional outcome of corpectomy in cervical spondylotic myelopathy

**DOI:** 10.4103/0019-5413.50855

**Published:** 2009

**Authors:** Kanishka E Williams, Rajesh Paul, Yashbir Dewan

**Affiliations:** Departments of Orthopaedics, Christian Medical College and Hospital, Ludhiana, Punjab, India; 1 Departments of Neurosurgery, Christian Medical College and Hospital, Ludhiana, Punjab, India

**Keywords:** Cervical corpectomy, cervical spondylotic myelopathy (CSM), cervical decompression, anterior approach

## Abstract

**Background:**

Cervical spondylotic myelopathy (CSM) is serious consequence of cervical intervertebral disk degeneration. Morbidity ranges from chronic neck pain, radicular pain, headache, myelopathy leading to weakness, and impaired fine motor coordination to quadriparesis and/or sphincter dysfunction. Surgical treatment remains the mainstay of treatment once myelopathy develops. Compared to more conventional surgical techniques for spinal cord decompression, such as anterior cervical discectomy and fusion, laminectomy, and laminoplasty, patients treated with corpectomy have better neurological recovery, less axial neck pain, and lower incidences of postoperative loss of sagittal plane alignment. The objective of this study was to analyze the outcome of corpectomy in cervical spondylotic myelopathy, to assess their improvement of symptoms, and to highlight complications of the procedure.

**Materials and Methods:**

Twenty-four patients underwent cervical corpectomy for cervical spondylotic myelopathy during June 1999 to July 2005.The anterior approach was used. Each patient was graded according to the Nuricks Grade (1972) and the modified Japanese Orthopaedic Association (mJOA) Scale (1991), and the recovery rate was calculated.

**Results:**

Preoperative patients had a mean Nurick's grade of 3.83, which was 1.67 postoperatively. Preoperative patients had a mean mJOA score of 9.67, whereas postoperatively it was 14.50. The mean recovery rate of patients postoperatively was 62.35% at a mean follow-up of 1 year (range, 8 months to 5 years).The complications included one case (4.17%) of radiculopathy, two cases (8.33%) of graft displacement, and two cases (8.33%) of screw back out/failure.

**Conclusions:**

Cervical corpectomy is a reliable and rewarding procedure for CSM, with functional improvement in most patients.

## INTRODUCTION

Cervical spondylotic myelopathy (CSM) is the most common cause of spinal cord dysfunction in older persons.^1^ Patients with CSM will generally have pain in the neck, subscapular area, or shoulder, numbness or paresthesias in the upper extremities, sensory changes in the lower extremities, motor weakness in the upper or lower extremities, gait difficulties, myelopathic or upper motor neuron and LMN findings. The upper motor neuron signs predominate typically below the level of the clinically expressed lesion.^2^

CSM usually develops insidiously. In the early stages neck stiffness is common because of the presence of advanced cervical spondylosis. Other common complaints include crepitus in the neck with movement; brachialgia, which is characterized as a stabbing pain in the pre or postaxial border of the arm, elbow, wrist, or fingers; a dull “achy” feeling in the arm; and numbness or tingling in the hands.

In patients who are mildly affected by CSM, a conservative approach can be taken. A variety of nonsurgical strategies have been used with variable success for the treatment of CSM. These include cervical traction, cervical immobilization (collar or neck brace), skull traction, and physical therapy.^3^

Once frank myelopathy occurs, surgical intervention is necessary. The primary goal of surgery is to decompress the spinal cord, thus giving the neural elements more room. Traditionally, cervical laminectomy, a posterior approach, has been used for surgical treatment of CSM. However, over the past 20 years, it has been increasingly recognized that laminectomy is not appropriate for all patients. Injury to the spinal cord, facet joint, dura, or nerve root can complicate the posterior cervical spine surgery, resulting in pain or deficit for the patient and occasionally requiring re-operation. For this reason, anterior approaches to the spine have been increasingly used.^4^

Ventral approaches to cervical decompressions include corpectomy and discectomy (multiple level) combined with osteophytectomy and/or discectomy. The use of single-level ventral decompression in the treatment of CSM is widespread. The rate of neurological improvement for patients with CSM undergoing corpectomy has been reported to be 39–83%.^5^,^6^ Compared to more conventional surgical techniques for spinal cord decompression, such as anterior cervical discectomy and fusion, laminectomy, and laminoplasty, patients treated with corpectomy have better neurological recovery, less axial neck pain, and lower incidences of postoperative loss of sagittal plane alignment.^7^ This study is an effort to evaluate the functional outcome of corpectomy in patients with cervical spondylotic myelopathy.

## MATERIALS AND METHODS

Twenty-four patients underwent corpectomy for spondylotic cervical myelopathy during June 1999 to July 2005, This is a six year study, five years being retrospective and 1 year prospective. The prospective study included cases from July 2004 to July 2005.

Consecutive patients with a definitive diagnosis of CSM were taken in this study. Patients presented with a variety of symptoms, such as numbness, clumsiness of the hands, neck pain, arm weakness, hand weakness, leg stiffness, loss of balance, and urinary urgency. Patients with a diagnosis of myelopathy due to other causes such as trauma, viral processes, inflammatory or autoimmune disorders, and tumors were excluded from the study.

Radiographs of the cervical spine (anteroposterior and lateral) were taken. MRI of the spine was not done in all cases. The most common level of involvement was C4-C5 (10 patients, 41.66%) followed by C3-C4 level (7 patients, 29.66%).C5-C6 level involvement was seen in three patients (12.50%), and four patients had multilevel involvement (16.67%). The mean number of vertebrae undergoing corpectomy was 1.3 (range, 1–3).

The patients were operated by a single surgeon. An anterior approach was used. Using fluoroscopy, the level of vertebra was identified. The surgeon then performed a discectomy at either end of the vertebral body (e.g., C4-C5 and C5-C6 to remove the C5 vertebral body), which was then removed using a burr. The defect was reconstructed with an autogenous iliac tricortical bone graft size, which depended upon the number of vertebrae removed. Out of 24 patients, 15 patients were instrumented using plates and screws. Wound was closed in layers and a cervical collar given. Drains were removed on the first day. In case of instrumented group, the patient was given a soft cervical collar for the first 5 days. Post operative motion exercises for neck were started at fifth day. The collar was worn intermittently and was discontinued after the graft was radiographically incorporated. In case of the uninstrumented group, a Philadelphia collar was given for 6 weeks followed by a soft collar for 6 more weeks. Intermittent mobilization was begun on the fifth postoperative day.

All the patients undergoing the study were followed up for a minimum period of 9 months postoperatively. The mean follow-up was 1 year (range, 8 months to 5 years). The patients were contacted through post, telephone, or personally, and their medical records were reviewed. All patients on follow-up were evaluated by a detailed clinical examination. Radiographs of the cervical spine (AP and lateral) were also taken. Patients in the retrospective group were also evaluated in a similar way. Each patient was graded according to the Nurick's Grade [Table 1] and the modified Japanese Orthopaedic Association (mJOA) Scale [Table 2]. As traditional values in our setup are different eg people do not usually eat with knife and fork, hence we modified the criteria of motor dysfunction score of the upper extremity by substituting the above symptoms as shown in Table 3. Functional outcome was assessed by analyzing the differences in pre and postoperative symptoms and signs of CSM and the Nurick's Grade and mJOA grade. The recovery rate was calculated as follows:

**Table 1:** Grading of cervical spondylotic myelopathy (Nurick, 1972)

Grade 0 - Signs or symptoms of root involvement without spinal cord disease
Grade I - Signs of spinal cord disease without difficulty in walking
Grade II - Difficulty in walking without effect on employment
Grade III - Difficulty in walking with effect on full-time employment
Grade IV - Can walk only with aid or walker
Grade V - Chair bound or bedridden

**Table 2:** Modified Japanese orthopaedic association grading for CSM (1991). Total score = 18

**I.**	**Motor dysfunction score of the upper extremity**	Grade
	Inability to move hands	0
	Inability to eat with spoon but able to move hands	1
	Inability to button shirt but able to eat with spoon	2
	Able to button shirt with great difficulty	3
	Able to button shirt with slight difficulty	4
	No dysfunction	5
**II.**	**Motor function score of the lower extremity**	
	Complete loss of motor and sensory function	0
	Sensory preservation without ability to move legs	1
	Able to move legs but unable to walk	2
	Able to walk on flat floor but with walking aid	3
	Able to walk up and/or down with handrail	4
	Moderate to significant lack of stability but able to walk up and/or downstairs without hand rail	5
	Mild lack of stability but walks with smooth reciprocation unaided	6
	No dysfunction	7
**III.**	**Sensory dysfunction score of the upper extremity**	
	Complete loss of hand sensation	0
	Severe sensory loss of pain	1
	Mild sensory loss	2
	No sensory loss	3
	Sphincter dysfunction score	
	Inability to micturate voluntarily	0
	Marked difficulty in micturation	1
	Mild to moderate difficulty in micturation	2
	Normal micturation	3

**Table 3:** Author's modification of Modified Japanese Orthopaedic Association Grading for CSM (1991)

**I.**	**Motor dysfunction score of the upper extremity**	Grade
	Unable to feed oneself	0
	Unable to button and unbutton his clothes, tie and untie the strings of his pajamas, clip his nails	1
	Able to button and unbutton his clothes, knot and unknot his pajamas, clip his nails with slight difficulty	2
	Able to button and unbutton his clothes, knot and unknot his pajamas, clip his nails with much difficulty	3
	Able to button and unbutton his clothes, knot and unknot his pajamas, clip his nails with great difficulty	4
	Normal	5
**II.**	**Motor function score of the lower extremity**	
	Complete loss of motor and sensory function	0
	Sensory preservation without ability to move legs	1
	Able to move legs but unable to walk	2
	Able to walk on flat floor but with walking aid	3
	Able to walk up and/or down with handrail	4
	Moderate to significant lack of stability but able to walk up and/or downstairs without hand rail	5
	Mild lack of stability but walks with smooth reciprocation unaided	6
	No dysfunction	7
**III.**	**Sensory dysfunction score of the upper extremity**	
	Complete loss of hand sensation	0
	Severe sensory loss of pain	1
	Mild sensory loss	2
	No sensory loss	3
	Sphincter dysfunction score	
	Inability to micturate voluntarily	0
	Marked difficulty in micturation	1
	Mild to moderate difficulty in micturation	2
	Normal micturation	3

$\text{Recovery rate = }\frac{\text{Postoperative mJOA score- Preoperative mJOA score }\times \text{ 100}}{18\text{ (total score)}-\text{Preoperative mJOA score}}$

Statistical analysis was done using Student's *t*-test and correlation analysis.

## Results

The mean age of patients suffering from CSM was 54 years (range, 35–72 years). Men were three times more affected than women (M:F ratio 3:1).

The mean duration of disease was 10.54 months (range, 5–48 months). Largely the patients had duration of disease less than six months (n = 16, 66.67%). Majority of the patients had an insidious onset of disease (n = 13, 54.17%). Preoperatively 13 patients (54.17%) had neck pain, 2 patients had headache (8.33%), and 2 patients (8.33%) had giddiness. Postoperatively on final follow-up one patient (4.17%) had neck pain, two patients had headache (8.33%), and no patient had giddiness. Preoperatively 19 out of 24 patients (79.17%) had pain radiating along upper limb, 16 patients (66.67%) had paresthesias along upper limb, 22 patients (91.67%) had weakness along upper limb, and 8 patients (33.33%) had fine motor function of hand. Postoperatively on final follow-up 4 out of 24 patients (16.67%) had radiating pain along upper limb, 4 patients (16.67%) had paresthesias along upper limb, 6 patients (25%) had weakness along upper limb, and 12 patients (50%) had fine motor function of hand. Preoperatively 14 out of 24 patients (58.33%) had paresthesias along lower limb, 21 patients (87.50%) had weakness of lower limbs, 20 patients (83.33%) had numbness of lower limbs, 6 patients (25%) had fasciculations of lower limb, and 20 patients (83.33%) had gait abnormalities.

Postoperatively on final follow-up, 8 out of 24 patients (33.33%) had paresthesias along lower limb, 11 patients (45.83%) had weakness of lower limbs, 5 patients (37.50%) had numbness of lower limbs, 5 patients (20.83%) fasciculations of lower limb, and 5 patients (20.83%) had gait abnormalities. Preoperatively 4 patients (16.67%) could comb their hair, 7 patients (29.17%) could make pieces of chapatti, 6 patients (25%) could button, 6 patients (25%) could open knots of pajama, and 5 patients (20.83%) could hold a pen. Postoperatively on final follow-up, 18 patients (75%) could comb their hair, 19 patients (79.17%) could make pieces of chapatti, 19 patients (79.17%) could button, 20 patients (83.33%) could open knots of pajama, and 21 patients (87.50%) could hold a pen. Preoperatively 19 out of 24 patients (79.17%) had sensory loss of upper limbs, and 17 patients (70.83%) had sensory loss of lower limbs. Postoperatively on final follow-up, 4 out of 24 patients (16.67%) had sensory loss of upper limbs and 5 patients (20.83%) had sensory loss of lower limbs.

The number of patients with a positive Babinski's reflex decreased postoperatively. Preoperatively 19 out of 24 patients (79.2%) had a positive Babinski's reflex. Postoperatively three patients (12.5%) had a positive Babinski's reflex. The number of patients with bladder dysfunction decreased from 4 (16.7%) preoperatively to none postoperatively.

The complications included one case (4.17%) of radiculopathy, two cases (8.33%) of graft displacement, two cases (8.33%) of screw back out/failure. There was also one case (4.17%) of vocal cord paralysis and two cases of respiratory compromise (8.33%).

Preoperative patients had a mean Nurick's grade of 3.83. Postoperatively patients had a mean Nurick's grade of 1.67. Preoperative patients had a mean mJOA score of 9.67. Postoperatively patients had a mJOA score of 14.50.

**Figure 1:**
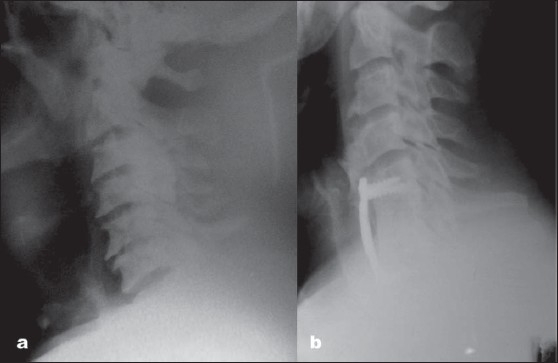
(a) X-ray cervical spine (lateral view) showing cervical spondylotic myelopathy in 43-year-old man with acute onset and rapid progression of symptoms. His preoperative Nurick's grade was 5 and mJOA score of 6; (b) Postoperative X-ray cervical spine (lateral view) showing corpectomy and anterior plate. His Nurick's grade was 1 and mJOA score was 17, with a recovery rate of 91.66%

**Figure 2:**
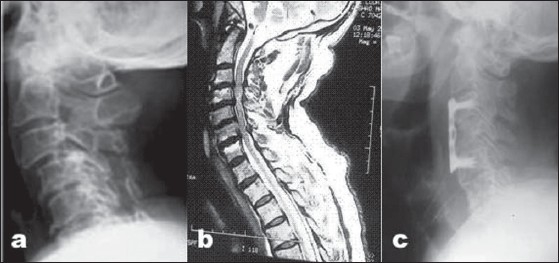
(a) X-rays cervical spine (lateral view) showing cervical spondylotic myelopathy (b) T2W MRI (saggital section) showing cervical spondylotic myelopathy post-operative (c) Post operative X-ray cervical spine showing corpectomy and anterior cervical plate

The mean recovery rate of patients postoperatively was 62.35% with SD of 32.82% [Table 4]. [Figures 1 and 2].

**Table 4:** Nurick's grade and mJOA score

	Preoperative		Postoperative	
		
	Mean (SD)	Range	Mean (SD)	Range
Nuricks grade	3.83 (1.37)	1–5	1.67 (1.83)	0–5
mJOA score	9.67 (3.05)	5–15	14.50 (3.56)	6–18

## DISCUSSION

Cervical spondylotic myelopathy is the most common cause of non-traumatic spastic paraparesis and quadriparesis. About 50%of people older than 50 years and 75% of those older than 65 years have typical radiographic changes of cervical spondylosis.^8^ About 23.6% of patients presenting with nontraumatic myelopathic symptoms had cervical spondylotic myelopathy in the United States.^9^

Spondylosis may lead to cord damage in three ways: there may be static-mechanical compression, dynamic-mechanical cord compression, and spondylotic changes may impair the circulation within the cord. Patients commonly become symptomatic when the cord is compressed by 30% or more.^10^ However, this varies considerably from patient to patient. Patients with severe canal stenosis are also at considerably increased risk of a major spinal cord injury with trauma.^11^

CSM presents in a highly variable manner. It usually manifests as one of five clinical syndromes depending on the anatomical location of the cord damage and the extent of disease. These are brachialgia and cord syndrome, central cord syndrome, anterior cord syndrome, Brown-Sequard syndrome, and transverse lesion syndrome.^12^

A full physical examination with a complete neurological assessment is needed for any patient with suspected CSM. On motor examination, mixed upper and lower motor neuron findings may be present in the upper extremity depending on the level of cord damage. CSM may cause decreased sensation of any or all modalities depending on the anatomic location of compression. Proprioceptive, vibratory and touch sensation may be impaired. Reflexes in the upper extremity may be hypo or hyperreflexive because both upper and lower motor neurons may be damaged. MRI remains the imaging modality of choice for CSM, even in an initial evaluation, because of its superior ability to show pathology of neural structures.^13^ MRI allows for clear visualization of cord impingement or compression and can be used to accurately measure space within the spinal canal.

Several conditions can mimic a myelopathy including cervical spondylotic neck pain or a radiculopathy, injuries or arthritis of the upper extremity, peripheral neuropathy or nerve injury, myopathies, vascular disease, drug intoxication or withdrawal, autoimmune diseases, and metabolic abnormalities.^14^ Once a diagnosis of CSM has been made, it may be very difficult to predict the disease course in a given patient. Somewhere between one-third and two-thirds of patients will deteriorate.^15^ The rest will stabilize, though significant improvement is rare.^15^

Nonsurgical therapy such as the use of NSAIDs, cervical collars, and steroids provides symptomatic relief in patients with CSM and may slow disease progression to some extent, though the latter statement has not been proven.^16^

Corpectomy has emerged as an excellent surgical modality CSM. In our study, the Nurick's Score improved from 3.83 to 1.67. In a study by Chagas *et al.*, the Mean Nurick Score improved from 2.97 to 2.1 after corpectomy.^17^ In a study by Rajashekar and Kumar, the mean Nurick score improved from 4.24 to 2.47 after corpectomy.^18^ The procedure of corpectomy increases mJOA score and thus the quality of life and activities of daily living.In our study, the increase was from preoperative score of 9.67–14.50. In a similar study by Chibbaro *et al.*, an improvement of mJOA score was seen from 12.2 to 15.^19^

Other surgical modalities of treatment of CSM are cervical laminectomy, laminoplasty, and discectomy. In a meta-analysis by Ratliff and Cooper, the mean recovery rate after cervical laminectomy and laminoplasty was reported to be 55% (range, 20–80%).^20^ Chiba *et al.* although reported good recovery rates after laminoplasty segmental motor paralysis, kyphosis, established before and after surgery, and late deterioration due to age-related degeneration remained challenging problems.^21^ Moreover, techniques in current use for expansive laminoplasty operations on the cervical spine damage the extensor mechanisms, resulting in restriction of neck motion, loss of lordosis, and persistent axial pains.^22^

Recovery rates in our study were 62.35% ± 32.82%. In a study by Sorar *et al.*, 85% of patients experienced a 50% or more recovery rate.^23^ Similarly Vyas *et al.*, have reported recovery rates of 66.9% following corpectomy.^24^

## CONCLUSION

Cervical spondylotic myelopathy can be treated effectively with gratifying results with corpectomy. The procedure has relatively better results when compared with other surgical modalities of treatment of CSM. However, patients need to be diagnosed at an early stage of the disease to have better results.
